# Biomechanical analysis and optimization of screw fixation technique for the cortical bone channel of lower thorax: Study protocol clinical trial (SPIRIT Compliant)

**DOI:** 10.1097/MD.0000000000019046

**Published:** 2020-02-14

**Authors:** Yang Yu, YiZhou Xie, Qiang Jian, Yin Shi, Guilong Zhang, Xiaohong Fan

**Affiliations:** a Hospital of Chengdu University of Traditional Chinese Medicine; b Chengdu University of Traditional Chinese Medicine, Sichuan Province, P.R.China.

**Keywords:** biomechanical analysis and optimisation, CBT screw, lower thoracic spine

## Abstract

Introduction: It is well known that the main segments of spinal fracture is thoracolumbar (T11-L11). Therefore, in addition to the lumbar, the lower thoracic vertebra (T9-T12) often has the clinical needs of implantation of cortical bone trajectory (CBT) screws. However, the anatomic parameters of the lower thoracic vertebrae are quite different from those of the lumbar vertebrae, which means that if CBT screws are to be implanted in the lower thoracic vertebrae, the selection of the screw entry point, the length, diameter, angle and path of the screws in each segment need to be redefined. Methods In this part, 3-dimensional finite element model was established to analyze the stress and fixation efficiency of CBT screws in thoracic vertebrae after 5000 times of fatigue loading of normal model and osteoporosis model. Discussion If the outcomes indicate the trial is feasible and there is evidence to provide some basic anatomical parameters for CBT screw implantation in the lower thoracic spine, so that the ideal insertion point, length, diameter, and angle of CBT screw in different segments of the lower thoracic spine were determined.

Trial Registration Chinese Clinical Trial Registry, ChiCTR1900026915.Registered on September 26, 2019.

## 1 Introduction

With the acceleration of the aging process and the increase of the number of patients with primary osteoporosis, there will be more and more patients suffering from it in the future.^[1]^ This means that the number of internal fixation screws that need to be implanted in the osteoporotic spine may also be increasing. Among all segments of the spine, the thoracolumbar segment (T11-L1) is the most prone area for spine fracture due to its high mobility.^[2–3]^

At present, thoracolumbar fracture is still the main type of posterior surgery, while the classic pedicle screw fixation technology plays an absolutely dominant role in the selection of posterior fixation. The technique of posterior transpedicular fixation of spine was used in clinic in 1970 s. At first, it was mainly used in the treatment of lumbar fracture. In view of its good fixation effect, it was gradually used in the reconstruction and fixation of various thoracic and cervical vertebrae. In the past decade, the technology has developed rapidly and has become the main means of spinal fixation.^[4–6]^

However, since the pedicle screw implantation path is from the posterior of the vertebral arch into the vertebral body, although three column fixation can be achieved from the posterior to the anterior, the screw path characteristics determine that the screw only has a good holding force in the pedicle with a limited length, while the vertebral body where the screw is implanted is mostly cancellous bone, so the holding force of the screw is relatively limited, and it is especially true for patients with osteoporosis.^[7–8]^

In the fixation of patients with severe osteoporosis, how to increase the holding power of screws is still a challenge for spinal surgeons. It has been reported that the length, diameter and thread design of screws can affect the biomechanical strength of screw fixation.^[9–12]^ Zindrick et al showed that increasing the length and diameter of the screw can indeed increase the biomechanical strength of the screw. However, he also stressed that increasing the length and diameter of the screw during the operation will inevitably lead to the risk of damaging the pedicle and the anterior and posterior walls of the vertebral body, and even the potential risk of damaging nerves and blood vessels.^[12]^ Another method which is widely used to increase the strength of pin bone interface fixation in patients with osteoporosis is to strengthen the nail way by using bone cement technology. Although bone cement strengthening technology can immediately increase the instant pull-out force of pedicle screws, the periodic anti flexion effect of screws after bone cement strengthening does not increase, and there is a risk of thermal injury and leakage of bone leading to nerve injury in the use process, and there is also a risk of pulmonary embolism in the operation process.^[13–14]^ In addition, some scholars have improved the design and implant technology of pedicle screw, and want to increase the screw holding force of osteoporosis patients by these methods, such as the design and application of porous screw and expansion screw, but the effect of these changes in osteoporosis patients is not satisfactory. The improvement of screw design and the use of strengthening materials are only focused on the improvement of traditional pedicle screw design and the change of screw placement technology, and these improved methods have not achieved good results.^[15–16]^

**Figure 1:**
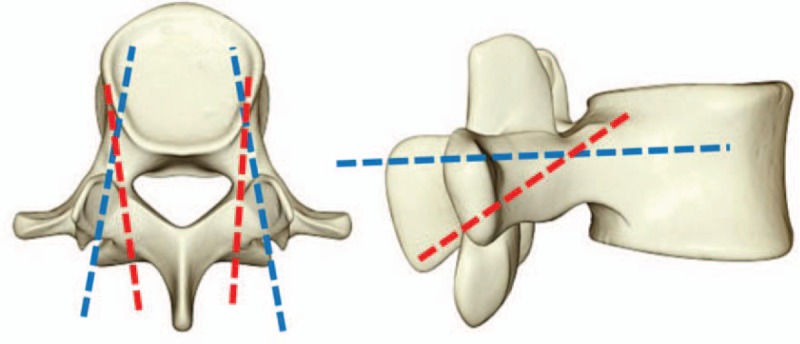
CBT screw versus pedicle screw fixation path (red: CBT screw; blue: pedicle screw). CBT = cortical bone trajectory.

In 2009, SANTONI et al put forward the internal fixation technology of cortical bone trajectory (CBT) through the improvement of screw implantation path and screw pathway. The screws used in CBT screw internal fixation technology are smaller in diameter and shorter in length than the traditional screws, but the thread arrangement is tighter, and fully contact with cortical bone concentration area, which can increase the strength of bone screw interface. Compared with the common pedicle screw, CBT screw has 4 significant advantages:

(1) The screw has four points of cortical fixation: lamina, vertebral body, internal, and external walls of pedicle, which makes the screw travel a long distance in cortical bone, and can significantly improve its holding power.
(2) In sagittal position, the screw is shaped from tail to head, and in transverse position, it is shaped from inside to outside, thus reducing the risk of nerve root injury and penetrating the inner wall of pedicle (see Fig. 1).
(3) The insertion point of screw is closer to the midline of vertebral lamina, which reduces the stripping of paravertebral muscles. Therefore, CBT screw technology is an excellent choice for patients with osteoporosis and screw failure requiring revision surgery. At the same time, CBT screw is a minimally invasive technology, which conforms to the development direction of spine surgery.^[17]^

Zhang et al compared the traditional pedicle screw with the cortical bone channel screw, and found that the single axial pull-out force of CBT was 30% higher than that of the traditional pedicle screw.^[18]^ Matsukawa et al evaluated the torque of the 2 screw techniques through in vivo experiments. The results showed that the torque of lumbar CBT screw fixation was 1.7 times higher than that of traditional pedicle screw.^[19]^ Gonchar et al prospectively compared the effect of lumbar CBT screw and percutaneous pedicle screw in posterior lumbar fusion, pointed out the occurrence rate of screw loosening and orthopedic maintenance effect of lumbar CBT screw technique. Results: the trauma caused by operation was better than that by percutaneous pedicle screw.^[20]^ Ueno et al thought that the difference of fixation strength of screws to vertebral body was mainly caused by different screw placement channels. They found that there was no significant difference in stiffness between 2 types of screws when using the same screw placement channel. However, no matter what type of screw was used in different screw placement channels, CBT technology had higher screw placement and pull-out force.^[21]^

At present, the basic and clinical research of CBT screw is mainly limited to the lumbar vertebra, and there is no relevant data research in China.^[22–23]^ However, the occurrence area of spine fracture is mostly in the thoracolumbar (T11-L1), which means that in the lower thoracic vertebra (T9-T12) patients with severe osteoporosis or revision surgery, CBT screws need to be inserted. However, there are great differences between the anatomical structure of the lower thoracic vertebrae and the lumbar vertebrae, and there are few studies on CBT screws of the lower thoracic vertebrae at home and abroad. Therefore, is it suitable to implant CBT screws in the lower thoracic vertebra of Chinese? We do not know the ideal point, the ideal path, the diameter range, the angle, the mechanical efficiency and the clinical reliability.

This trial aims at the main clinical problems of spinal surgery, that is, the fixation efficiency of traditional pedicle screw may be insufficient in the case of osteoporosis thoracolumbar fracture, and there is a clinical demand for CBT screw implantation in the lower thoracic vertebra. Although CBT screw has a certain mechanical advantage for the fixation efficiency of osteoporosis patients, at present, the relevant research at home and abroad is mainly limited to the lumbar spine, and there are few reports about the clinical anatomy parameters, fixation efficiency and in vivo work of CBT screw in the lower thoracic spine in China.^[22–23]^

## 2 Objections

This study will improve the clinical anatomy and biomechanical study of CBT screw in the lower thoracic vertebrae (T9-T12) of different groups in China, and provide the corresponding theoretical basis and basis for its clinical application. It provides a new choice for patients with osteoporosis who may need fixation in thoracolumbar region.

## 3 Methods

### 3.1 Clinical anatomy of CBT screws in the lower thoracic spine of Chinese

The computed tomography (CT) images of the lower thoracic vertebrae (T9-T12) of 40 male and 40 female normal adults in China were collected. The trial was examined and approved by the Ethics Committee of the Hospital of Chengdu University of traditional Chinese medicine. No. nt-6354

### 3.2 Applicants inclusion criteria

The age of applicants is between 20 to 60 years old in good health and they are good at spirit and intelligence. They obey the arrangement of the research group, accept the treatment plan designed by the research group and sign the informed consent;

### 3.3 Applicants exclusion criteria

Participants suffered from severe spinal degeneration or severe irreversible damage of multiple spinal columns such as spinal tuberculosis and tumor.

### 3.4 Determination of the ideal driving point

#### 3.4.1 Determination of the transverse axis of the screw

**Figure 2:**
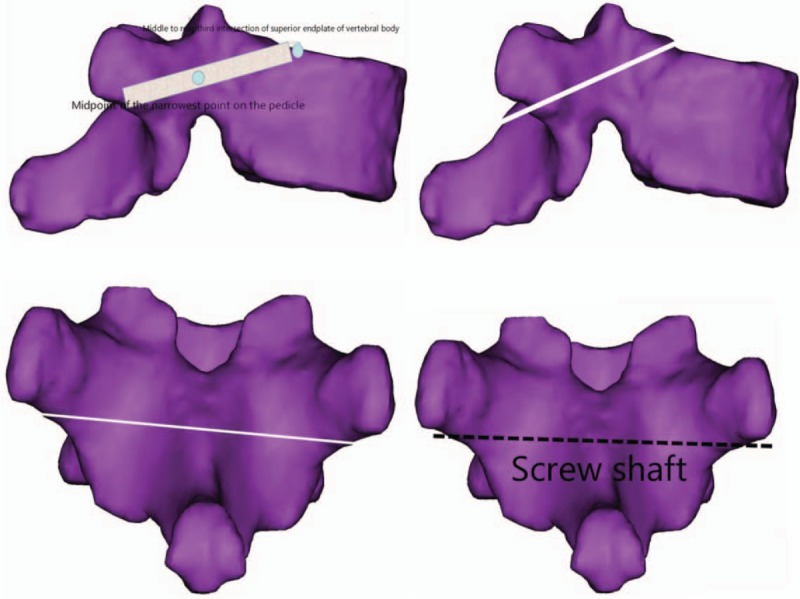
Schematic diagram of horizontal axis of screw.

Simulate the routing of the screw with diameter of 3.0 mm on the lateral image of the vertebral body. The screw passes through the middle point of the narrowest point of the pedicle height from the bottom to the back one thirds of the junction area of the superior endplate of the vertebral body, and make a oblique upward transverse section along the bilateral axis. The intersection line between the section and the posterior lamina of the vertebral body is the transverse axis of the screw (see Fig. 2).

#### 3.4.2 Determination of the longitudinal axis of the screw

**Figure 3:**
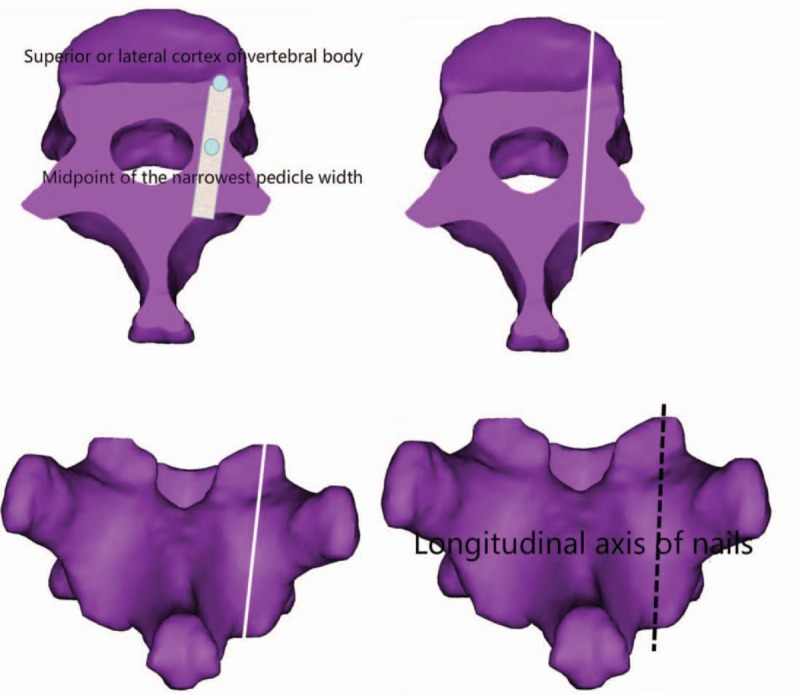
Longitudinal axis of screw.

Simulate the routing of the screw with diameter of 3.0 mm on the above oblique cross-section. The screw passes through the middle point of the narrowest part of the pedicle from the inside to the outside and ends at the superior part of the vertebral body or the lateral cortex. Make a sagittal section along this axis. The intersection of this section and the posterior vertebral plate is the longitudinal axis of the screw (see Fig. 3).

#### 3.4.3 Determination of the insertion point

**Figure 4:**
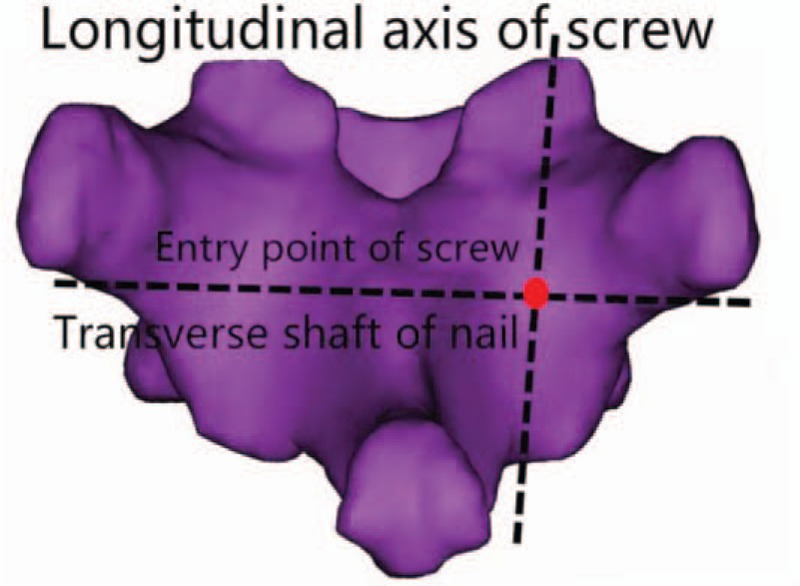
Schematic diagram of nailing point.

The intersection of the above transverse and longitudinal axes is the ideal insertion point of CBT screws. Carefully observe the distribution of the ideal insertion point of each vertebra of the lower thoracic vertebra (T9-T12) in 40 men and 40 women, and the relationship between the ideal insertion point and the surrounding anatomical structure, so as to determine the ideal insertion point of CBT screws in different segments of the lower thoracic vertebra (see Fig. 4).

### 3.5 Determination of measurement plane

#### 3.5.1 Transverse section

**Figure 5:**
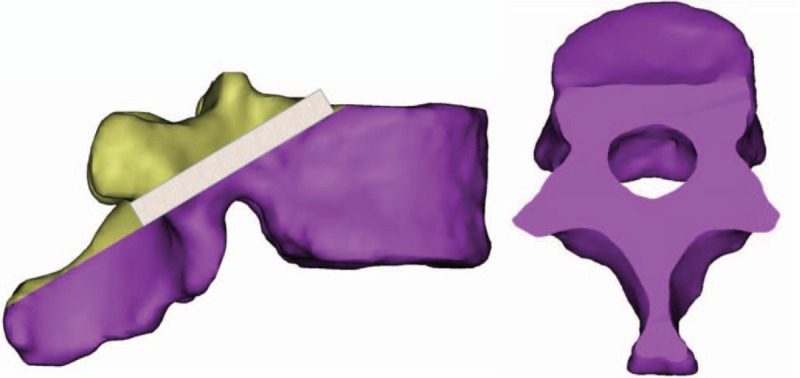
Schematic diagram of cross section.

The CBT screw is placed at the ideal point of insertion according to the above experiment, and the screw runs from the bottom to the top, passing through the middle point of the narrowest point of pedicle height, and ends at the superior endplate. The cross section is made according to the above-mentioned screw track for the measurement of transverse section (see Fig. 5).

#### 3.5.2 Sagittal section

**Figure 6:**
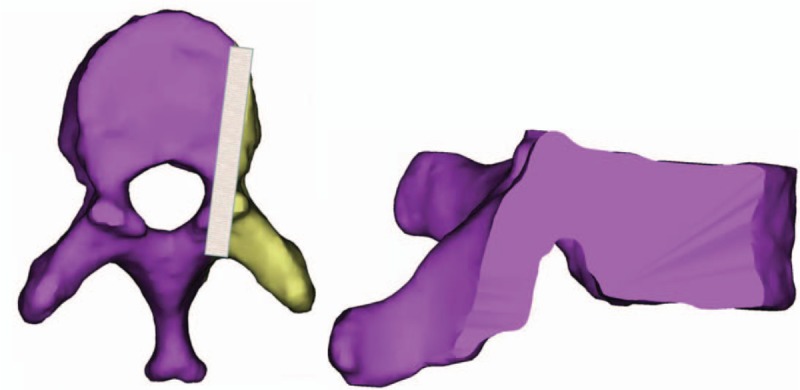
Schematic of sagittal section.

CBT screws are placed according to the ideal screw entry point obtained from the above experiments. The screws run from the inside to the outside and pass through the middle point of the narrowest point of pedicle width and end at the superior or lateral cortex. Sagittal section is made according to the above-mentioned screw path for sagittal correlation measurement (see Fig. 6).

### 3.6 Data collection and analysis

#### 3.6.1 Measurement parameters of related nailways

**Figure 7:**
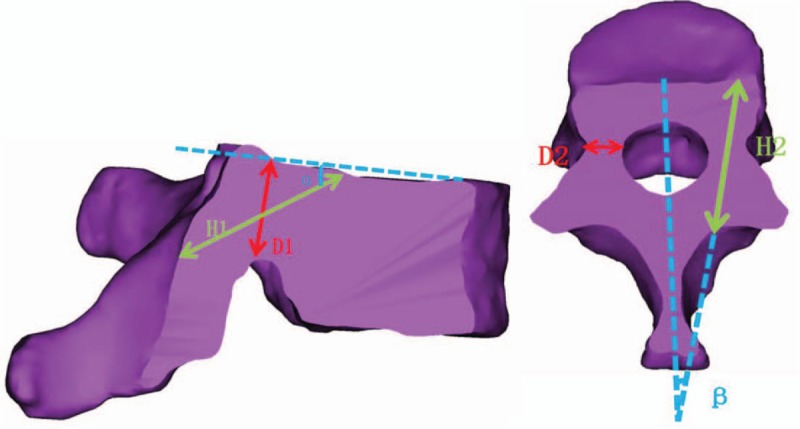
Measurement diagram of related anatomical parameters.

According to the requirements of CBT screw related screw placement technology, the relevant parameters of anatomical measurement are designed on the above-mentioned cross-section and sagittal section (see Table 1 and Fig. 7).^[17]^ The corresponding anatomical parameters were measured and counted on the 3-dimensional model of the lower thoracic vertebrae, and the anatomical differences between men and women and different segments were analyzed. The ideal length, diameter and angle of CBT screws in different vertebrae of lower thoracic vertebrae were analyzed by using the measured anatomical data and freeform computer-aided software, so as to design a suitable model for the placement of CBT screws in order to improve the accuracy of the placement of CBT screws in Chinese lower thoracic vertebrae. Ethics committee will monitor the whole procedure including data gathering.

**Table 1:**
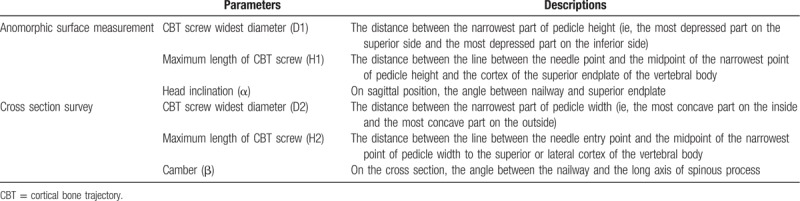
Measurement of anatomical parameters of lower thoracic vertebra (T9-T12) (left/right sides).

#### 3.6.2 Three dimensional finite element analysis of CBT screw fixation technique of lower thoracic spine in Chinese

The meticulous CT images of the lower thoracic vertebrae of healthy adults and the elderly patients with osteoporosis were collected. The 3-dimensional model of the lower thoracic vertebrae was reconstructed by means of mimics13.0 (Materialise, Belgium). After the mesh was divided, the 3-dimensional finite element model of the normal lower thoracic vertebrae and the osteoporosis lower thoracic vertebrae were established by ansys15.0 finite element software (ANSYS). In ansys15.0, the 3-dimensional finite element model of the lower thoracic vertebrae of healthy adults and the 3-dimensional finite element model of the lower thoracic vertebrae of osteoporosis were tested according to the experimental flow chart shown in Table 2. Single segment (T10-T11) and double segment (T9-T11) were fixed respectively. Pedicle screw and CBT screw fixation instruments were assembled into the above model respectively, and the 2 models were analyzed and fixed in 2 different ways. When the device is fixed, the stress and fixation efficiency of pedicle screw and CBT screw after 5000 times of vertical fatigue at 3 Hz are studied to explore the biomechanical principle and efficiency of CBT screw fixation of lower thoracic vertebra.

**Table 2:**
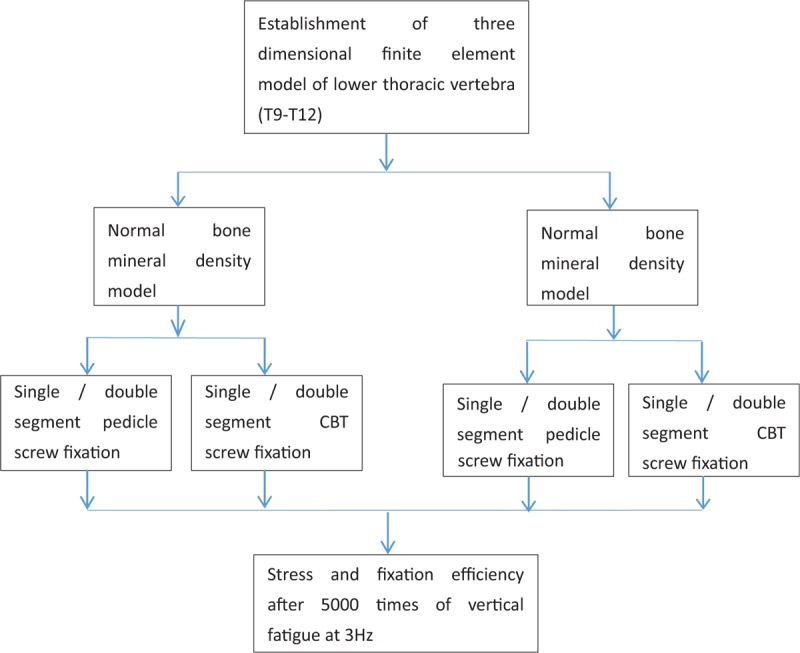
flow chart of 3D finite element analysis.

#### 3.6.3 Data management

The investigators and research assistants in the study team will collect the data and enter them directly into the electronic-Case Report Form. The data will be securely stored, with specific access rights granted to members of the study team according to their role in the study. Participants, healthcare professionals, the public, and other relevant groups could get the results of the trial as soon as we completes the trial and publishes our achievements.

### 3.7 Safety

Any observed side effects related to radiation exposure will be documented throughout the trial period and reported to the Sponsor without delay. These data will be provided for periodical review by the Data and Safety Monitoring Board.

## 4 Discussion

Based on the previous research in the field of clinical applied anatomy and medical biomechanics, this trial measures the anatomical data related to the CBT screws of the lower thoracic spine of Chinese men and women, improves and optimizes the screw placement path and method of the CBT screws of the lower thoracic spine of Chinese people, and objectively analyzes the fixation technology of the CBT screws of the lower thoracic spine in the normal vertebral body and the clinical application value of osteoporotic vertebral body.

Its innovation lies in that this anatomical measurement is made in accordance with the actual direction of the screw, no matter the transverse section or the sagittal section. The measured screw length, width and angle are all actual values, which is more accurate and reliable. This method avoids the problem that the measured length of the nailway is not its real length, but the projection length of the nailway in the horizontal plane or the sagittal plane caused by the previous study of the measurement of the nailway.

This study also provides a technical choice for patients with osteoporosis who need posterior fixation of the lower thoracic spine, and makes a detailed anatomical, biomechanical and in vivo simulation study on this technical choice.
